# Cognitive bias modification for paranoia (CBM-pa): a randomised controlled feasibility study in patients with distressing paranoid beliefs

**DOI:** 10.1017/S0033291722001520

**Published:** 2023-07

**Authors:** Jenny Yiend, Charlene L. M. Lam, Nora Schmidt, Bryony Crane, Margaret Heslin, Thomas Kabir, Philip McGuire, Christopher Meek, Elias Mouchlianitis, Emmanuelle Peters, Daniel Stahl, Antonella Trotta, Sukhwinder Shergill

**Affiliations:** 1 Institute of Psychiatry, Psychology and Neuroscience at King's College London, London, UK; 2 The State Key Laboratory of Brain and Cognitive Sciences, The University of Hong Kong, Hong Kong; 3 Laboratory of Clinical Psychology and Affective Neuroscience, The University of Hong Kong, Hong Kong; 4 The McPin Foundation, London, UK; 5 South London & Maudsley NHS Foundation Trust, London, UK; 6 Kent and Medway Medical School, Canterbury, UK

**Keywords:** Cognitive bias modification, interpretation bias, paranoia, psychosis, RCT

## Abstract

**Background:**

Cognitive Bias Modification for paranoia (CBM-pa) is a novel, theory-driven psychological intervention targeting the biased interpretation of emotional ambiguity associated with paranoia. Study objectives were (i) test the intervention's feasibility, (ii) provide effect size estimates, (iii) assess dose–response and (iv) select primary outcomes for future trials.

**Methods:**

In a double-blind randomised controlled trial, sixty-three outpatients with clinically significant paranoia were randomised to either CBM-pa or an active control (text reading) between April 2016 and September 2017. Patients received one 40 min session per week for 6 weeks. Assessments were given at baseline, after each interim session, post-treatment, and at 1- and 3-months post-treatment.

**Results:**

A total of 122 patients were screened and 63 were randomised. The recruitment rate was 51.2%, with few dropouts (four out of 63) and follow-up rates were 90.5% (1-month) and 93.7% (3-months). Each session took 30–40 min to complete. There was no statistical evidence of harmful effects of the intervention. Preliminary data were consistent with efficacy of CBM-pa over text-reading control: patients randomised to the intervention, compared to control patients, reported reduced interpretation bias (*d* = −0.48 to −0.76), improved symptoms of paranoia (*d* = −0.19 to −0.38), and lower depressed and anxious mood (*d* = −0.03 to −0.29). The intervention effect was evident after the third session.

**Conclusions:**

CBM-pa is feasible for patients with paranoia. A fully powered randomised control trial is warranted.

Interpretation bias refers to ‘a consistent tendency to interpret emotionally ambiguous stimuli, situations, or events in a negative (or positive) manner’ (Lee, Mathews, Shergill, & Yiend, 2016). Interpretation biases are one of the cognitive processes described by the threat anticipation cognitive model of persecutory delusions (Freeman, Garety, Kuipers, Fowler, & Bebbington, 2002). In this model when a patient experiences something that they consider unusual or odd (an anomalous experience) this leads them to search for a meaning or explanation of that experience, which in turn can lead to the formation of a threat belief. This belief can be maintained by cognitive processes, one of which is biased interpretations. The selective processing of the negative or paranoid meanings of ambiguous information (i.e. the interpretation bias) will exacerbate patients' subjective perception of, and their actual exposure to, information that confirms their threat beliefs and the expectation of harm.

In line with this theoretical model, negative interpretation bias is proposed as a causal factor in the development and maintenance of paranoia in healthy, subclinical and clinical populations. Healthy individuals with high trait paranoia have been shown to interpret ambiguous information related to paranoid content more negatively compared to individuals with low trait paranoia (Savulich, Freeman, Shergill, & Yiend, 2015). This negative bias is evident before the onset of disorder in individuals at risk of developing psychosis (Yiend et al., 2019) and is seen at various points in the illness trajectory (Savulich, Shergill, & Yiend, 2017). Most convincingly from a causal perspective, manipulating paranoid interpretation bias produced similar changes in how distressed participants were when hearing ambiguous laughter and whether they believed the laughter was directed towards themselves (Savulich et al., 2020). Taken together, these studies suggest that interpretation biases play a potent role in paranoia.

Existing psychological interventions such as cognitive therapy may change the underlying biases associated with paranoia, promoting more adaptive cognitive well-being. Yet the Schizophrenia Commission (2012) reported that cognitive behavioural therapy is received by only one in 10 of those who could benefit in the UK and has shown only moderate effect sizes for delusions (Mehl, Werner, & Lincoln, 2015; Van der Gaag, Valmaggia, & Smit, 2014). Other psychological treatments available for psychosis include Cognitive Remediation Therapy, which has only limited effects on symptoms (Wykes, Huddy, Cellard, McGurk, & Czobor, 2011), and Metacognitive Training (Moritz & Woodward, 2007), which has small to moderate effects on positive symptoms (Eichner & Berna, 2016; Philipp et al., 2019). Thus, more accessible and targeted interventions are warranted. Cognitive Bias Modification (CBM) is a theory-driven, cognitive intervention that may be able to fill the existing service gap. CBM works by manipulating biases toward more adaptive processing in emotionally ambiguous situations. Since its inception, it has been employed as an adjunct intervention for various mental disorders, including anxiety (Hallion & Ruscio, 2011; Woud, Verwoerd, & Krans, 2017) and depression (Jones & Sharpe, 2017). However, its appropriate adaption for paranoia and resulting efficacy in the psychosis population is yet to be examined.

We are aware of only two studies to date that have employed CBM-I in patients with psychosis. Steel et al. (2010) targeted comorbid anxiety, rather than paranoia specifically, using a CBM method designed for anxiety. No significant changes were observed in either interpretation bias or state anxiety. Turner et al. (2011) reported significant improvements in social anxiety after manipulating interpretations of socially ambiguous scenarios. In contrast, the present study was designed to modify the paranoia-relevant interpretation bias that directly reflects the paranoid beliefs associated with psychosis, as shown in previous basic experimental research in high trait (Savulich et al., 2015), patient (Savulich et al., 2017) and prodromal (Yiend et al., 2019) samples.

For the present study we created a customised version of CBM-I, labelled the ‘Cognitive Bias Modification for paranoia’ (CBM-pa), that targets the specific cognitive mechanisms contributing to paranoia as identified in our previous basic research studies (Savulich et al., 2015, 2017; Yiend et al., 2019). In our earlier report we applied a single-session of CBM-pa to individuals with high levels of trait paranoia and found preliminary evidence that paranoid interpretation biases could be manipulated using CBM-pa (Savulich et al., 2020). The double-blind, randomised controlled trial reported here was designed to further investigate the feasibility and preliminary efficacy of CBM-pa for individuals with persistent, distressing paranoid symptoms. In line with the intended purpose of feasibility trials (Eldridge et al., 2016), the main objectives of this study were:

- test the feasibility of CBM-pa including rates of recruitment, dropout, and follow-up, randomisation, as well as the length of the protocol and its safety;
- provide initial estimates of the effects of CBM-pa on interpretation bias, clinical outcomes (symptoms of paranoia, depression and anxiety) and vulnerability to stress/distress;
- assess the dose–response relationship of CBM-pa;
- select primary and secondary outcomes for future full clinical trials.

## Methods

### Design

**Fig. 1.**
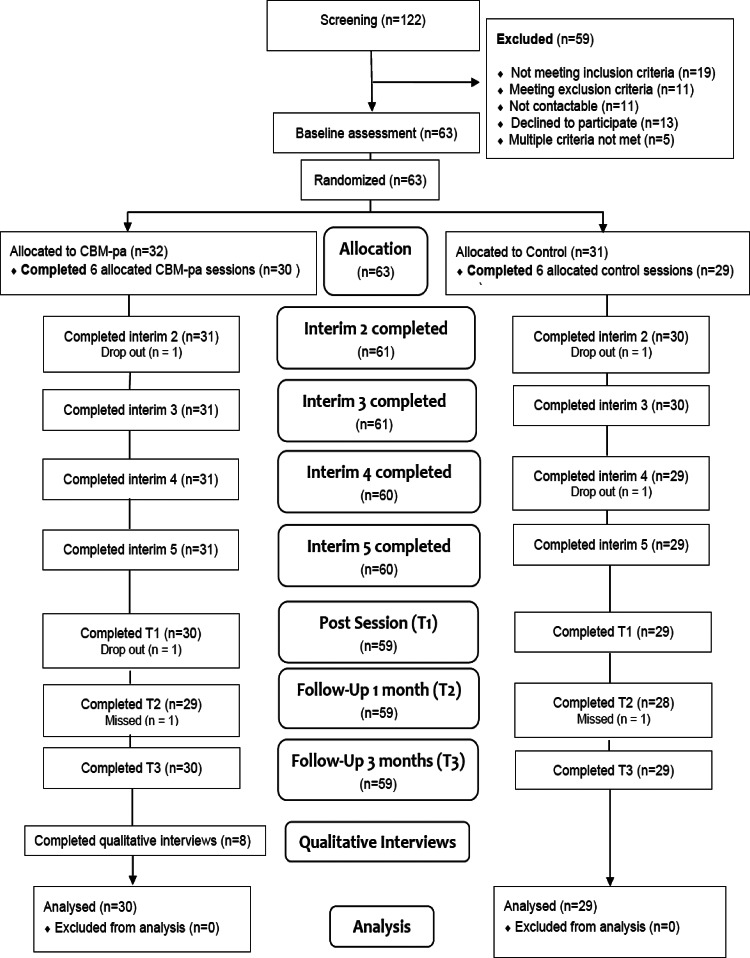
CBM-pa CONSORT diagram. *Note*: ‘Completed’ defined as participant attended the session and at least one data point was acquired; ‘Drop out’ defined as participant no longer participating in the trial; ‘Missed’ defined as participant did not attend session but returned for further sessions. Adapted from CONSORT 2010 Flow Diagram (CONSORT: TRANSPARENT REPORTING OF TRIALS).

The study was a single-site, feasibility, double-blind, two-arm randomised controlled trial. Participants were randomised into either the CBM-pa group (*n* = 32) or control group (*n* = 31) after the baseline assessment at a 50:50 ratio (Fig. 1).

### Participants

A total of 122 individuals with clinically significant persecutory or paranoid symptoms were recruited from the Institute of Psychiatry, Psychology and Neuroscience clinical research registers; the South London and Maudsley NHS Trust research registers, including a ‘Consent 4 Contact’ scheme and the McPin Foundation charity service user networks between April 2016 and September 2017. In line with good practice recommendations for pilot studies (Lancaster, Dodd, & Williamson, 2004) no formal power calculations were performed. The recruitment target was 60 randomised patients, which is within the recommended range for providing robust variance estimates for effect size estimation (Browne, 1995; Kieser & Wassmer, 1996). The inclusion and exclusion criteria were detailed in Supplementary Text. The final sample consisted of 63 participants (mean age = 45.6 years, s.d. = 10). The CONSORT diagram is shown in Fig. 1.

### Interventions

#### CBM-pa intervention

The intervention was intended to train individuals to process ambiguity in a more helpful way by first reading text inviting paranoid interpretations and then generating responses reflecting an alternative, non-paranoid interpretation. Since there was evidence of content specificity of the training items, i.e. interpretation bias was only found for material permitting paranoid interpretations rather than other ambiguous materials (Savulich et al., 2017), we maximised the clinical relevance of the training items of CBM-pa by developing over 100 examples of everyday situations drawn from the experiences of the Lived Experience Advisory Panel (LEAP) (Yiend et al., 2014, 2017).

**Fig. 2.**
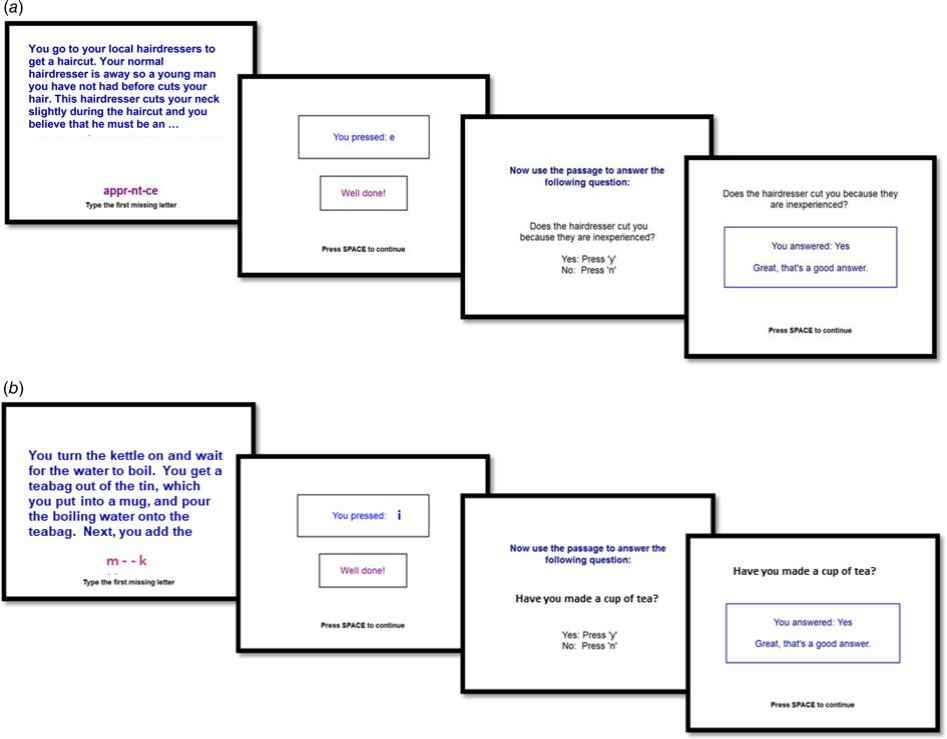
(a) Example of a CBM-pa intervention item: an intervention item presents an ambiguous situation in the trial format shown. In this example a common interpretation is that the cut was accidental, but many patients with paranoid symptoms would interpret the cut as deliberate. Each intervention trial uses a word task and question to guide patients into making helpful, rather than paranoid, interpretations. In this example, if participants enter the wrong letter they are given a ‘clue’ (more letters appear) and if still wrong, the correct completed word is shown. Likewise, the question is carefully constructed such that it always reinforces the non-paranoid interpretation of the preceding passage (the cut is the result of inexperience). To answer the question ‘correctly’ participants must endorse the non-paranoid meaning and cannot continue until they do so. If the ‘wrong’ answer is given a feedback message indicates ‘that is one answer, is there another possibility?’ (b) Example of an active control (neutral text-reading) item: a control item is identical in format and manner of delivery to an intervention item, the only difference is the textual content and its meaning. Control items relay factual information which is unambiguous and emotionally neutral. The word completion and question remains neutral and factual.

An example of one item is illustrated in Fig. 2a. In this example a common interpretation is that the cut was accidental, but many patients with paranoid symptoms would interpret the cut as deliberate. The intervention uses the word completion task and the follow-up question to guide patients into making the more helpful interpretation of the scenario, as opposed to their more usual interpretation. Basic research shows that passive exposure to items (e.g. just reading) is ineffective; trials are constructed to require active engagement in processing the alternative, non-paranoid meaning of each passage. This is done using the word completion and questions. The word completion is constructed such that it always resolves the ambiguity of the preceding passage into the benign, non-paranoid interpretation (e.g. an apprentice is inexperienced and therefore likely to make accidental cuts). Likewise, the question is constructed such that it always reinforces the non-paranoid interpretation of the preceding passage (the cut is the result of inexperience). To answer the question ‘correctly’ participants must endorse the non-paranoid meaning and cannot continue until they do so.

The training items covered six categories relevant to paranoia: physical harm, social/interpersonal threat, medical/paramedical/health care threat, threat of persecution/spying, delusions of reference/magical thinking and other general suspiciousness/distrust. In the present trial, CBM-pa consisted of 40 items per session and was delivered once per week over six consecutive weeks.

#### Active control

The active control program was identical in every way to the intervention save for the content of individual items and was designed to control for non-specific treatment effects, as well as the passage of time (Mohr et al., 2009). Non-specific treatment effects included contact with researchers, human interaction variables such as empathy, attention and so on, participants' outcome expectations and procedural elements such as reading passages of text, inputting responses and time spent on the computer. The content of each control item was unambiguous and emotionally neutral; thus, it did not involve any active resolution of an emotionally ambiguous situation. All items were reviewed by the LEAP to ensure their neutrality and readability. Each item followed an identical format, trial design and procedure to those given in the intervention (Fig. 2b). Items described everyday tasks or situations that relayed factual information in six categories: home, leisure, knowledge, transport, sports and media. Similar to CBM-pa, 240 control items were divided into six sessions of 40 items each.

#### Blinding

Researcher blinding was implemented by the creation of unique, randomly labelled computer programmes which masked the assigned treatment arm from researchers. As CBM-pa is self-administered researchers could remain blind by ensuring they did not observe the computer screen after set-up was complete. Participants in both conditions were told they were randomly assigned to either intervention or control groups.

### Measures

As the study was a feasibility evaluation of the CBM-pa intervention, we collected three domains of possible outcome measures (interpretation bias, clinical symptoms and stress/distress) to further select primary and secondary outcomes for future full clinical trials. The SRT, PANSS item 6 and Virtual Reality Environment task were designated as the provisional primary outcomes in each respective domain. A variety of measures were given and one selected in each domain on the basis of the following parameters: sensitivity to change and effect sizes, acceptability to patients, feasibility of administration (e.g. length, response options, mode of administration) and opinion of the Trial Steering Committee on presentation of the final results. Measures were selected based on our previous study (Savulich et al., 2015) with goals to replicate sample characteristics and associated findings in this clinical population.

#### Measures of feasibility

Feasibility of CBM-pa was assessed through rates of recruitment, dropout, follow-up, randomisation, protocol length and its safety. The feasibility of randomisation was also evaluated via (i) the proportion of patients willing to be randomised and (ii) the integrity of double-blinding. First, researchers would record any instances of inadvertent unblinding during the experiment. Second, participants were asked to guess in which condition they were in after the intervention. Third, participants rated the expectation of benefits using the Credibility and Expectancy Questionnaire (Devilly & Borkovec, 2000), which assessed how much they felt the intervention had helped reduce their paranoia symptoms and other related symptoms, and how much improvement in the symptoms had occurred on a nine-point Likert scale ranging from 1 = *not at all* to 9 = *very much*.

To measure the potential harm of reading about potentially paranoid situations, a series of Visual Analogue Scales (VAS) items were employed to assess the immediate impact on state anxiety, sadness, paranoia and friendliness after taking part in the session.

#### Measure of bias

We measured interpretation biases by two well-established, reliable and valid cognitive experimental measures, namely the Scrambled Sentences Task and Similarity Rating Task.

*Scrambled Sentences Task* (SST; Rude, Wenzlaff, Gibbs, Vane, & Whitney, 2002, 2003). It is a robust measure of interpretation bias that requires participants to re-order five out of six words to create a grammatically correct sentence (e.g. ‘are me hostile strangers to friendly’). Participants had 5 min to complete as many of the 15 sentences as possible (defined as the variable B below). Before unscrambling the 15 sentences, participants were asked to memorise a six-digit number (e.g. 615239) for a later recall test and keep it in mind while completing the task. Each word string can be unscrambled into sentences with either paranoid or non-paranoid meaning. Paranoid and non-paranoid interpretation bias scores were calculated as a percentage of either paranoid or non-paranoid (respectively) interpretations made. Bias scores therefore range from 0 to 100 with higher scores reflecting larger bias favouring the specified direction of interpretation (paranoid or non-paranoid). Full scoring instructions used are available at https://osf.io/neuza/. These SST bias scores have good reliability (Cronbach's *α* = 0.73; Smith et al., 2018)

*Similarity Rating Task* (SRT; Eysenck, Mogg, May, Richards, & Mathews, 1991). The SRT was adapted from Mathews and Mackintosh (2000) and Eysenck et al. (1991) and developed with paranoia-relevant content (Savulich et al., 2015). Items in the SRT described ambiguous scenarios that permitted either paranoid- or non-paranoid interpretations which allowed participants to make their own spontaneous interpretations. It consisted of two parts. Participants first read 12 ambiguous passages, followed by an assessment of their spontaneous interpretation on each previously presented ambiguous passage. Participants were given the title of the previous passage as a reminder and, four sentences, presented one at a time. Two of the sentences were possible paranoid (T−) and non-paranoid (T+) interpretations of the original passage and labelled ‘targets’. A further two sentences labelled ‘foils’ presented paranoid (F−) and non-paranoid (F+) content unrelated to the corresponding passage, and were control items used to measure response bias. Participants were asked to rate ‘how similar in meaning is this sentence to the passage you saw earlier?’ using a 1 ( = *very different in meaning*) to 4 ( = *very similar in meaning*) scale. Items were presented in random order within each task and at a pace determined by the participant to allow for different reading and comprehension speeds. Interpretation bias scores range from +3 to −3. Higher scores indicated stronger paranoid bias in the present study. The SRT bias score had acceptable reliability (Cronbach's *α* = 0.63; Smith et al., 2018)

#### Measures of demographics and primary diagnoses, clinical symptoms and traits

The following assessments measured demographics, primary diagnoses, clinical symptoms and traits – the details of the specific scales are presented in the Supplementary materials. Demographics comprised the variables listed in Table 1. Primary diagnosis was assessed using the Structured Clinical Interview for DSM-5 – Research Version (SCID-5-RV). Clinical outcomes are listed in Table 2 and measured with the following assessments: *Paranoia Scale* (PS; Fenigstein & Vanable, 1992), *The Green et al Paranoid Thoughts Scale* (GPTS; Green et al., 2008), *Positive and Negative Symptom Scale* (PANSS; Kay, Fiszbein, & Opler, 1987), *The Peters et al Delusion Inventory* (PDI-21; Peters, Joseph, Day, & Garety, 2004), *Hospital Anxiety and Depression Scale* (HADS; Zigmond & Snaith, 1983), *Cognitive Flexibility Scale* (CFS; Martin & Rubin, 1995). Stress/Distress (Table 2) was measured with the *Laughter task* (Green et al., 2011), *Virtual Reality Environment* (VRE) and *State Social Paranoia Scale* (SSPS; Freeman et al., 2007).

**Table 1.** Demographic characteristics and primary diagnoses of overall sample (*N* = 63)

Variable		CBM-pa group (*n* = 32)		Control group (*n* = 31)		Combined (*N* = 63)	
		*n*	%	*n*	%	*N*	%
*Age*	Mean (SD)	30	43.7 (9.80)	31	47.5 (10.0)	61	45.6 (10.0)
*Gender*	Male	22	68.8%	20	64.5%	42	66.7%
	Female	10	31.3%	11	35.5%	21	33.3%
*Ethnicity*							
	White	16	51.6%	14	22.2%	30	47.6%
	Mixed and other	1	3.2%	5	7.9%	6	9.5%
	Asian	2	6.5%	3	4.8%	5	7.9%
	Black	13	41.9%	8	12.7%	21	33.3%
*Education level*							
	No qualifications	9	28.1%	8	26.7%	17	27.4%
	GCSE/O’ levels	5	15.6%	5	16.7%	10	16.1%
	A levels	3	9.4%	3	10.0%	6	9.7%
	Vocational/college	4	12.5%	10	33.3%	14	22.6%
	University/professional qualification	11	34.4%	4	13.3%	15	24.2%
*Employment*							
	Unemployed	25	78.1%	23	0.7%	48	76.2%
	Student	1	3.1%	0	0.0%	1	1.6%
	Full time	3	9.4%	1	3.2%	4	6.4%
	Part time	3	9.4%	7	22.6%	10	15.9%
*Dyslexia*	(Self-reported, yes/no)	4	13.3%	11	35.5%	15	24.6%
*Relational status*							
	Alone	17	53.1%	19	63.3%	36	58.1%
	Alone, with children	3	9.4%	4	13.3%	7	11.3%
	Partner/spouse/children	4	12.5%	4	13.3%	8	12.9%
	Parents, other family, friends and other	8	25.0%	3	10.0%	11	17.7%
*SCID-5 RV primary diagnosis*							
	Schizophrenia	20	66.7%	19	61.3%	39	63.9%
	Schizophreniform disorder	1	3.3%	0	0%	1	1.6%
	Schizoaffective disorder	2	6.7%	3	9.7%	5	8.2%
	Major depression with psychotic symptoms	4	13.3%	5	16.1%	9	14.8%
	Bipolar disorder with psychotic symptoms	1	3.3%	2	6.5%	3	4.9%
	Anxiety disorder with psychotic symptoms	2	6.7%	2	6.5%	4	6.6%

SCID-5 RV, Structured Clinical Interview for DSM-5 – Research Version (First et al., 2015).

**Table 2.** Baseline-adjusted pairwise comparisons between CBM-pa group and control group at each time point (T1: post-intervention, T2: 1-month and T3: 3-month follow-up)

Measure	Time	Mean difference	(95% CI)	*z*	*p*	%
Interpretation bias						
Similarity Rating Task Target: bias	T1	−0.24	(−0.55 to 0.06)	−1.55	0.120	–
	T2	−0.29	(−0.61 to 0.02)	−1.82	0.069	–
	T3	−0.39	(−0.70 to −0.08)	−2.43	**0.014**	**–**
Similarity Rating Task Foil: bias	T1	−0.10	(−0.30 to 0.11)	−0.96	0.339	–
	T2	−0.11	(−0.32 to 0.10)	−1.04	0.298	–
	T3	−0.09	(−0.29 to 0.12)	−0.84	0.399	–
Scrambled Sentences Task: paranoid bias	T1	−13.11	(−27.99 to 1.77)	−1.73	0.084	–
Scrambled Sentences Task: non-paranoid bias	T1	24.13	(11.30–36.95)	3.69	**0.000**	**–**
Cognitive flexibility	T1	0.58	(−6.50 to 7.67)	0.16	0.872	1.2%
Clinical outcomes						
PANSS: item 6	T1	−0.11	(−0.76 to 0.54)	−0.33	0.740	−2.1%
	T2	−0.20	(−0.86 to 0.45)	−0.60	0.547	−3.9%
	T3	−0.43	(−1.08 to 0.22)	−1.31	0.191	−8.4%
Paranoia Scale	T1	−4.03	(−10.52 to 2.47)	−1.22	0.224	−6.4%
	T2	−6.72	(−13.29 to −0.15)	−2.01	**0.045**	−10.7%
	T3	−3.37	(−9.80 to 3.06)	−1.03	0.304	−5.4%
Paranoid Thoughts Scale: total	T1	−7.95	(−22.80 to 6.89)	−1.05	0.294	−8.9%
	T2	−9.02	(−23.97 to 5.94)	−1.18	0.237	−10.1%
	T3	−3.04	(−17.63 to 11.53)	−0.41	0.683	−3.4%
Peters Delusions Inventory	T1	0.60	(−1.55 to 2.74)	0.54	0.587	6.2%
	T3	−0.80	(−3.11 to 1.52)	−0.68	0.500	−8.3%
HADS: total score	T1	−2.03	(−4.79 to 0.72)	−1.45	0.148	−28.9%
	T2	−1.12	(−3.91 to 1.66)	−0.79	0.429	−16.0%
	T3	−0.18	(−2.96 to 2.60)	−0.13	0.900	−2.5%
Stress/distress						
VRE pre–post change: sadness	T1	−4.30	(−15.30 to 6.70)	−0.77	0.443	**–**
VRE pre–post change: paranoia	T1	−1.77	(−14.13 to 10.60)	−0.28	0.779	**–**
VRE pre–post change: friendly	T1	−0.04	(−0.17 to 0.09)	−0.63	0.527	**–**
VRE pre–post change: anxiety	T1	0.26	(−13.87 to 14.39)	0.04	0.971	**–**
VRE SSPS	T1	0.06	(−0.50 to 0.63)	0.21	0.830	**–**

HADS, Hospital Anxiety and Depression Scale; PANSS, Positive and Negative Symptom Scale; SSPS, State Social Paranoia Scale; VRE, virtual reality environment.

A positive value of mean difference means that the participants of the CBM-pa group score higher than the participants of the control group. For clinical outcomes, the change of percentages from mean at baseline is provided.

### Procedure

#### Ethics

The study was approved by the London – City Road and Hampstead Research Ethics Committee on 26 February 2016 (reference: 16/LO/0071). Informed written consent was obtained from all participants. Selected participants were randomly assigned to either CBM-pa or active control program using a computer-generated randomisation formula.

#### Randomisation

Double-blind randomisation was conducted by King's College London's Clinical Trials Unit. A web-based randomisation system was designed, using the bespoke King's Clinical Trials Unit (KCTU) randomisation system. The randomisation system was created in collaboration with the trial analyst (DS) and the chief investigator (JY) and maintained by KCTU for the duration of the project. It was hosted on a dedicated server within King's. System access was strictly restricted through user-specific passwords to the authorised research team members. Participant initials and age were entered on the randomisation system. Randomisation was undertaken by researchers going to www.ctu.co.uk and clicking the link to access the randomisation system. A full audit trail of data entry was automatically date and time stamped, alongside information about the user making the entry within the system. No data could be amended in the system; however, researchers could request KCTU to add notes against individual subject entries to clarify data entry errors. Upon request, KCTU provided a copy of the final exported dataset to the CI in .csv format and the CI will onward distribute as appropriate. Randomisation was at the level of the individual using the method of minimisation with fixed block sizes stratified by gender (male, female), severity of baseline paranoia according to the PANSS item 6 score (3; 4–7) and severity of interpretation bias measured by the SRT screening bias score (above or below −0.7)

#### Trial procedure

Participants received one 40 min session per week, over 6 weeks at King's College London and were assessed at four timepoints, T0 (baseline), T1 (post-intervention), T2 (1-month follow-up), and T3 (3-month follow-up), by trained research workers, who were blind to treatment group. Before the intervention (T0), sociodemographic information, biases (SRT, SST), clinical symptoms and traits (PANSS item 6, PS, GPTS, PDI-21, HADS) and cognitive flexibility (CFS) were measured. Immediately following training (T1), the second SRT and SST, counterbalanced from baseline, were given to measure post-intervention bias, followed by the Laughter Task and VRE. Clinical symptoms and traits (PANSS item 6, PS, GPTS, PDI-21, HADS) and CFS were assessed again at this timepoint. To understand the dose–response relationship, biases and paranoid beliefs were assessed using an 8-item SRT (using target items only) and PANSS-item 6 after each interim session (a total of four times between T0 and T1). Both groups continued to receive Treatment as Usual (TAU), which comprised individualised combinations of medication and care coordination.

### Statistical analysis

Data from all participants who entered the study were included in the analysis. Feasibility of trial procedures was examined using proportions and exact Clopper Pearson's 95% confidence intervals for assessments of feasibility in terms of rates of recruitment, dropout and follow-up at 1- and 3-months.

In line with the intended purpose of feasibility trials (Eldridge et al., 2016), formal analyses were not conducted; analyses reported here are for interest only and no firm conclusions on efficacy can be drawn regardless of significance level. To provide the initial estimates of the effects of CBM-pa, we ran a random-effects model with various clinical outcomes as the dependent outcome. Independent categorical variables were time with three categories (post-intervention, 1-month and 3-month follow-up), treatment arm (CBM-pa or control) and the interaction between groups and time. Baseline values of the outcome were included as a covariate to control for potential baseline differences. To account for the repeated observations over time, we included participant number as a random factor.

Dose–response effects were assessed by measuring paranoid symptoms (PANSS item 6) and interpretation bias (SRT) at baseline and after each session. Dose–response analyses were computed similarly to random-effects analyses but without baseline as a predictor because the measures were all taken after randomisation.

Given the small size of the sample employed, statistical analyses were corrected for multiple testing by using a reduced *α* level of 0.01. Analyses were conducted using STATA 15 (StataCorp, 2013). Standardised effect sizes (Cohen's *d*) with 95% confidence intervals were presented, which Cohen cautiously interpreted as *d* = 0.20, small effects; *d* = 0.50, medium effects; and *d* = 0.80, large effects (Cohen, 1992). Since the study was not powered for testing differences between groups, emphases were placed on the confidence interval of effect size estimates rather than the *p* values.

## Results

### Demographics

Sixty-three participants took part in the study. Means and standard deviations of the sample demographic characteristics are shown in Table 1. The mean age of all participants was 45.6 years (s.d. = 10.00), ranging from 23 to 63 years. Most participants were male (66.6%), unemployed (76.2%) and lived alone (58.1%). Means and standard deviations for males and females separately by arm for each time point are presented in the Supplementary materials. An overrepresentation of males in the sample was expected given the gender imbalance commonly observed in those suffering psychosis (Aleman, Kahn, & Selten, 2003).

### Feasibility

A full list of the recruitment sources and their relative success rates is provided in online Supplementary Table S1. The largest proportion of the sample came from the team's research registers (41%), around a quarter of the sample came from the McPin charity collaborators and a further quarter via self-referral following study publicity. A total of 122 individuals were screened, among which 59 (48.4%) were excluded. The recruitment rate was 51.2%. Two participants from each group dropped out during the intervention, leading to an overall rate of completion of 93.7%. Follow-up rates were high: 90.5% (*n* = 57) and 93.7% (*n* = 59) attended the 1- and 3-month follow-up assessment, respectively (online Supplementary Table S2).

All participants agreed to blind randomisation with no concerns raised. Neither researchers nor participants reported any instance of inadvertent unblinding during the study. Participants' guesses regarding intervention condition were at chance level: 12 out of 27 participants (55.6%) of the control group guessed that they were in the treatment group compared to 14 out of 29 participants (48.3%) of the CBM-pa group, χ^2^(1) = 0.30, *p* = 0.57. Both groups reported similar levels of expectations regarding whether the intervention had (i) improved their levels of paranoia (*d* = −0.02, 95% CI −1.09 to 1.03), (ii) helped to reduce their symptoms (*d* = −0.15, 95% CI −0.80 to 0.20) and (iii) their paranoia symptoms had been reduced (*d* = −0.24, 95% CI −1.15 to 0.2). These small effect sizes provided support for the feasibility of double-blind randomisation of CBM-pa.

Our analyses showed that there were no significant changes in the state sadness, paranoia or friendliness assessed by the VAS across the four interim sessions in both groups. These results suggested that reading scenarios about potentially paranoia-inducing situations in CBM-pa did not create a negative or harmful mood.

#### Preliminary treatment effects: interpretation bias

**Fig. 3.**
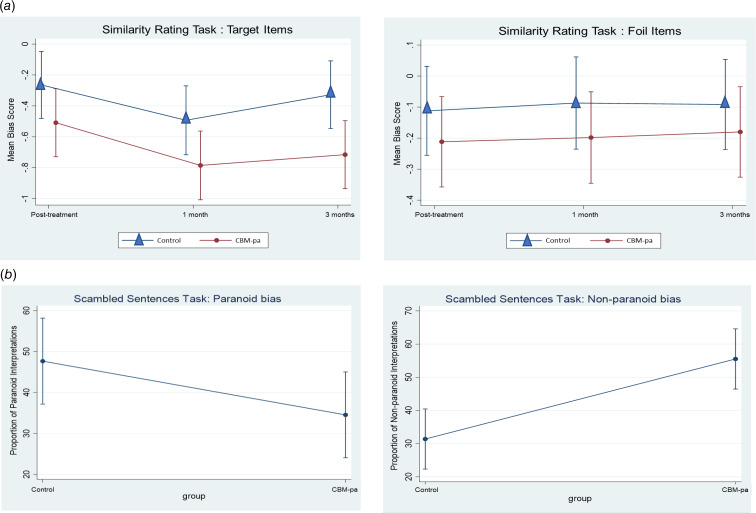
Bias measures and baseline-adjusted change over time in the: (a) target items and foil items of the Similarity Rating Task; (b) paranoid interpretations and non-paranoid interpretations of the Scrambled Sentences Task. Error bars represent the 95% confidence interval of the mean values.

Pairwise comparisons between CBM-pa group and control group at T1 (post-treatment), T2 (1-month follow-up) and T3 (3-month follow up) for all outcome measures are presented in Table 2. The standardised effect sizes (Cohen's *d*) and their confidence intervals at each time point are illustrated in Table 3. As shown in Fig. 3b participants in the CBM-pa group were, on average, 0.51 standard deviations lower in paranoid-related bias measured by the SST compared to the control counterparts at post-intervention. Change on the non-paranoid bias score is also shown. The reduction in paranoid-specific interpretation bias was also consistently observed in the SRT across the three time points comparing the two groups (Fig. 3). The differences between the two groups on cognitive flexibility were comparable at post-intervention (Table 2).

**Table 3.** Standardised effect of CBM-pa group compared to control group on outcome measures at each time point (T1: post-intervention, T2: 1-month and T3: 3-month follow-up).

Measure	Time	Cohen's *d*	95% CI
Interpretation bias			
Similarity Rating Task Target: bias	T1	−0.48	−2.13 to 0.25
	T2	−0.57	−2.33 to 0.09
	T3	−0.76	−2.68 to −0.30
Similarity Rating Task Foil: bias	T1	−0.23	−1.35 to 0.47
	T2	−0.25	−1.43 to 0.44
	T3	−0.20	−1.31 to 0.52
Scrambled Sentences Task (SST): paranoid bias	T1	−0.51	−2.14 to 0.13
Scrambled Sentences Task (SST): non-paranoid bias	T1	0.94	0.86– 2.81
Cognitive flexibility	T1	0.08	−1.66 to 1.96
Clinical outcomes			
PANSS: item 6	T1	−0.11	−1.52 to 1.08
	T2	−0.21	−1.72 to 0.91
	T3	−0.44	−2.16 to 0.43
Paranoia Scale	T1	−0.23	−1.18 to 0.28
	T2	−0.38	−1.49 to −0.02
	T3	−0.19	−1.10 to 0.34
Paranoid Thoughts Scale: total	T1	−0.22	−1.24 to 0.38
	T2	−0.25	−1.31 to 0.32
	T3	−0.08	−0.96 to 0.63
Peters Delusions Inventory	T1	0.11	−0.56 to 0.99
	T3	−0.15	−1.12 to 0.55
HADS: total score	T1	−0.29	−1.33 to 0.20
	T2	−0.16	−1.09 to 0.46
	T3	−0.03	−0.82 to 0.72
Stress/distress			
VRE pre–post change: sadness	T1	−0.18	−1.24 to 0.54
VRE pre–post change: paranoia	T1	−0.07	−1.09 to 0.82
VRE pre–post change: friendly	T1	0.00	−0.01 to 0.01
VRE pre–post change: anxiety	T1	0.01	−0.97 to 1.01
VRE SSPS	T1	0.06	−0.49 to 0.59

HADS, Hospital Anxiety and Depression Scale; PANSS, Positive and Negative Symptom Scale; SSPS, State Social Paranoia Scale; VRE, Virtual Reality Environment.

#### Preliminary treatment effects: clinical outcomes

**Fig. 4.**
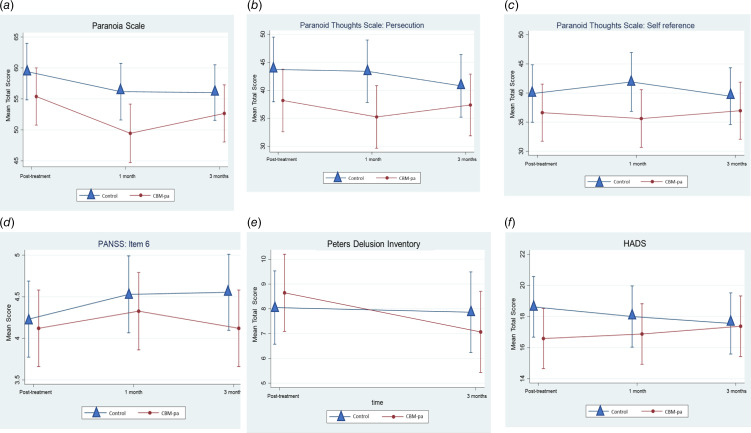
Baseline-adjusted change over time in clinical outcomes (a) Paranoia Scale, (b) Green et al Paranoid Thought Scales – persecutory subscale, (c) Green et al Paranoid Thought Scales – self reference subscale, (d) PANSS – item 6, (e) Peters et al Delusions Inventory, and (f) Hospital Anxiety and Depression Scale. Error bars represent 95% confidence interval of the mean values.

The baseline-corrected changes in the clinical outcomes across time points and groups are presented in Fig. 4 and Table 3.

#### Preliminary treatment effects: vulnerability to stress/distress

On the laughter task, 28 participants (49.1%) noticed the laughter. Among those who noticed the laughter, participants in the control group tended to score higher than the CBM-pa group in rating ‘something about you’ (*d* = −0.31, 95% CI −0.83 to 0.22). In the virtual reality environment, the changes in state paranoia measured by the SSPS and the state sadness, paranoia, friendliness and anxiety measured by the VAS were generally small at post-intervention in both groups (Table 2).

#### Dose–response relationship

**Fig. 5.**
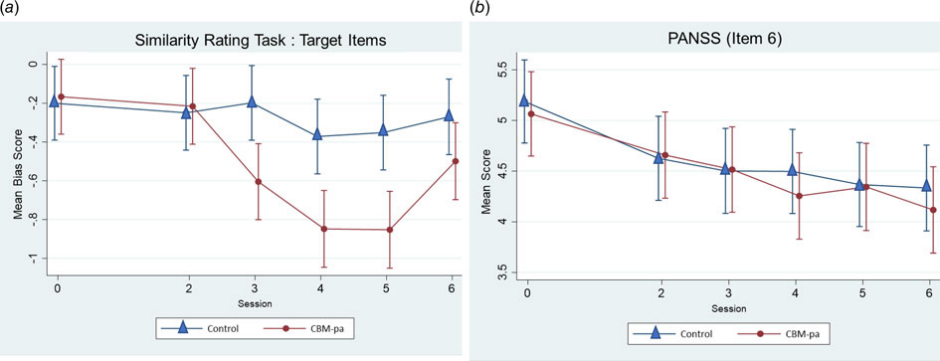
(a) Dose–response curve for interpretation bias. The figure shows the mean interpretation bias score (95% confidence intervals) at T0 (before the first session, dose = 0) and after sessions 2–6 (dose = 2–6) for each treatment arm. (b) Dose–response curve for PANSS item 6. The figure shows the mean PANSS item 6 score (95% confidence intervals) at T0 (before the first session, dose = 0) and after sessions 2–6 (dose = 2–6) for each treatment arm.

The *time* *×* *session* interaction revealed that participants in the CBM-pa group reported fewer paranoid interpretations from Session 3 onwards (*p* < 0.001), suggesting that the target mechanism had changed after three sessions (see Fig. 5a). There were significant differences between the two groups with CBM-pa scoring significantly higher at times 2, 3, 4 and 5 (all *p* < 0.01). The difference became smaller and non-significant at time 6 due to an increase from time 5 to 6 in the control arm (*p* = 0.001) while there were little changes in the CBM-pa arm (*p* = 0.44.).

The dose–response relationship was not evident on the PANSS (Fig. 5b). There were no significant *time* *×* *session* interaction effects across sessions in both groups, *p* = 0.91.

## Discussion

The findings of the trial are promising, both in terms of the feasibility and possible benefits of the intervention for reducing interpretative bias and symptoms of paranoia and related symptoms in psychosis patients. It demonstrated preliminary evidence of possible treatment effects on both the target mechanism and some clinical outcomes, although not powered to detect these.

### Feasibility of the intervention

Recruitment to the study ran smoothly. The overall recruitment rate was 51.2%. This estimate permits informed calculations about the referral rate required in order to achieve a specified sample size at full trial. We concluded that the trial was feasible on the basis of high completion rates (>90%), low dropout (<5%), high follow-up rates (>90%), the acceptability of randomisation, absence of evidence of any harmful effects on state mood and practicality of the protocol as delivered. Patients were not aware of which intervention they had received, therefore minimising the potential placebo effect. Reading about potentially paranoid situations in the CBM-pa condition did not appear to exacerbate negative mood over the course of the intervention, nor did it pose any risk of harm to patients with distressing paranoia. Additionally, the acceptability of CBM-pa has been evaluated qualitatively in a separate report (Leung et al., 2019). The feedback was generally positive and encouraging, and the intervention was acceptable in patients with psychosis.

### Initial estimates of the effect of CBM-pa

In line with the purpose of feasibility studies, this study was not powered to detect treatment effects. Nevertheless the pattern of data was consistent with treatment effects immediately after the intervention that favoured the CBM-pa intervention over the neural text-reading control program. The standardised effect sizes were medium to large (*d* ranging from −0.48 to −0.76) on measures related to interpretation bias. Participants receiving the CBM-pa intervention reported less paranoid ideation at post-intervention and at the two follow-ups and the standardised effect sizes ranged from small to medium (*d* ranging from −0.19 to −0.38). These effect sizes were comparable to other studies integrating the training of biases into cognitive behavioural therapy (*d* = 0.38 in Waller et al., 2015; *d* = 0.36 in Garety et al., 2015). Improvement in symptoms of anxiety and depression was also observed immediately following CBM-pa with an effect size of 0.29. Therefore our findings are consistent with a recent study reporting the use of cognitive bias modification for interpretation to improve symptoms of anxiety and paranoia (Hurley, Hodgekins, Coker, & Fowler, 2018).

Although effects of CBM-pa on paranoia symptoms at follow-ups were present on some measures, they appeared to wane over time, being generally weakest at 3 months. This pattern was fairly consistent across paranoid symptom measures. The Paranoia Scale showed the most robust changes (e.g. *mean difference* = −6.72 at 1-month follow-up, *p* = 0.045) and appeared to be the most sensitive measure to the effects of CBM-pa. The GPTS and PANSS (item 6, clinician-rated) showed similar patterns of paranoia reduction, but generally weaker effects. In contrast the estimated effects on depression and anxiety symptoms appeared smaller, dropping from −0.29 at post-intervention to near negligible −0.03 at 3-month follow-up. This is perhaps not surprising given that CBM-pa was designed to target paranoia specifically, whereas other forms of CBM have been developed to combat depression/anxiety (Yiend et al., 2014). In future the combination of different CBM versions targeting different symptoms and/or mechanisms might be a fruitful avenue to investigate (Leung, Yiend, & Lee, 2022; Leung, Yiend, Trotta, & Lee, 2022). The estimates of population variances of the main outcomes for future power calculations are presented in online Supplementary Table S4.

### Dose–response relationship

Due to practical constraints only one mechanistic measure (SRT) and one clinical measure (PANSS item 6) could be administered after each session, nor was the study sufficiently powered to detect session by session effects on either outcome. Nevertheless, intervention effects upon the target mechanism were evident after the third session and peaked at the fifth session, suggesting patients began to spontaneously select more adaptive patterns of interpretation after three training sessions. Notwithstanding small reductions after sessions 4 and 6, there was no convincing pattern of change on the clinical measure. It will be important in future work to investigate the optimum dosage of CBM-pa by conducting a Phase II dose finding study. One possible design would be to establish the minimum number of sessions required to achieve an a priori-defined clinically significant reduction of paranoid symptoms using retrospective dose–response modelling (Voils et al., 2014).

### Selection of primary outcomes for future trial

Following the presentation and subsequent discussion of the data presented in this paper, the Trial Steering Committee concluded as follows regarding the most suitable primary outcomes to use in a future clinical trial. For the target mechanism the SRT was the preferred primary outcome because of the strong evidence of sensitivity to change and good effect sizes within the present data. The SST, despite larger effect sizes post-treatment, lacked data at interim and follow-up and was therefore recommended to be administered regularly in future as a secondary outcome. As an inclusion criterion, PANSS item-6 appeared to perform well; it was quick and simple to administer as a screening instrument and allowed a degree of clinical judgement to be brought to bear on participants' suitability to take part, which would not be possible if using a self-report screening measure. However, as a primary outcome measure the PANSS item 6 proved unsuitable. Despite evidence suggesting that single item measures can be valid both in general (Bergkvist, 2015; Bergkvist & Rossiter, 2007) and in psychiatric samples (e.g. Coffino, Grilo & Udo, 2020), in this study this item was found to be insensitive to any effects of the intervention and impractical to administer repeatedly. However, using the PANSS item 6 as a secondary outcome could still be considered. Instead a self-report measure was preferred, with the Paranoia Scale selected as the most suitable due to its good effect sizes, status as a standardised scale used as a primary outcome elsewhere and being short and easy to use. The GPTS and HADS should be retained as secondary outcomes given the observed effects and their practicality, whereas the PDI was long with lesser effects and should be dropped. The transferrable effect of CBM-pa to paranoia-inducing situations in a virtual reality environment was not readily observed in the present trial. Although the Laughter Task showed some sensitivity to patients' real-life responses to stress it could be administered only once, was resource intensive to deliver and required face-to-face contact with participants, all of which made it unsuitable for use in a large-scale trial. Instead a trial might consider including user-endorsed recovery measures designed to capture any wider, daily-functioning benefits known to be important to patients' themselves (e.g. Neil et al., 2009).

### Limitations

First, patients in this study were recruited from a single clinical service unit in the UK, which may not be representative of a wider patient population. Future studies could consider using randomised, multi-site controlled trials. Second, we excluded patients who were receiving or had received psychotherapy in the past 3 months to avoid possible confounds in our analyses. Future studies may consider including these participants to examine the potential synergistic effect of combining psychotherapy and CBM-pa in modulation of bias and mood symptoms. Third, our measures of the mechanism of action of the intervention (interpretation bias) were not designed to be ‘process pure’. Although we had two measures of bias to provide convergent validity and the tasks chosen had established sensitivity in this patient population to interpretation biases associated with paranoia, we make no strong claims based on the current data about this mechanism of action. Fourth, our choice of a non-specific treatment control (Mohr et al., 2009) – reading passages of neutral, unambiguous text – presents some limitations. This controlled for factors including contact with researchers, human interaction variables (empathy, attention, etc.), participants' outcome expectations and procedural elements (reading passages of text, inputting responses and time spent on the computer). However, group differences could have been the result of exposure to ambiguous information, emotional information or both, rather than the modification of interpretation bias *per se*. Nevertheless, a frequent choice of control condition in feasibility trials is the ‘no treatment’ control (e.g. waitlist) or ‘treatment as usual’, since the avoidance of Type II errors is deemed to be the priority (Mohr et al., 2009). Thus our use of an active, non-specific treatment control was considerably more conservative and more rigorous than usual. Finally, our sample was diagnostically heterogeneous because CBM-pa is a transdiagnostic intervention designed to target distress caused by paranoid symptoms rather than treating any specific diagnosis. This approach avoids the use of stigmatizing labels, reflected the expressed preferences of our service user group and is grounded in current psychopathology research that advocates the identification and treatment of underlying functional mechanisms (Sauer-Zavala et al., 2017). Nevertheless, the present results may have been influenced by diagnosis and it will be important in future work to ascertain whether treatment response is moderated by diagnostic category.

## Conclusions

The study established quantitative feasibility information, provisional treatment effects and variance estimates, preliminary dose information, and suitable primary outcomes for evaluation of CBM-pa in a fully powered future trial using a double-blind randomised controlled design. A fully powered randomised controlled trial to test treatment efficacy appears warranted.

## Supplementary material

For supplementary material accompanying this paper visit https://doi.org/10.1017/S0033291722001520. click here to view supplementary material

## Data access and responsibility

The principal investigator had full access to all of the data in the study and takes responsibility for the integrity of the data and the accuracy of the data analysis.

## Trial registration

The CBM-pa is registered at the Current Controlled Trials ISRCTN: 90749868.
