# Supplementary material for: Cognitive bias modification for paranoia (CBM-pa): a randomised controlled feasibility study in patients with distressing paranoid beliefs
Source: Psychol Med. 2022 Jun 14;53(10):4614–26. doi: 10.1017/S0033291722001520 (PMC10388312; doi:10.1017/S0033291722001520)
Supplement: Supplementary file 1 [file S0033291722001520sup.zip › S0033291722001520sup001.docx]

Table S1. Recruitment Sources and Success Rates

| Source | Number Screened | Number Consented | Success Rate | % of final sample |
| --- | --- | --- | --- | --- |
| Shergill’s Research Register | 32 | 24 | 0.75 | 38.1 |
| McPin charity | 41 | 16 | 0.39 | 25.4 |
| Self-referral (publicity, snowballing etc) | 23 | 15 | 0.65 | 23.81 |
| NHS Trust Consent for Contact scheme | 5 | 4 | 0.8 | 6.35 |
| Peters' research register | 9 | 2 | 0.22 | 3.17 |
| Other/Unknown | 12 | 2 | 0.17 | 3.17 |
| *Totals* | ***122*** | ***63*** |  | ***100*** |

Table S2. Follow up rates of each group

|  | Total | % (95% C.I.) | CMB-pa | % (95% C.I.) | Control | % (95% C.I.) |
| --- | --- | --- | --- | --- | --- | --- |
| Baseline | 63 |  | 32 |  | 31 |  |
| T1 | 62 | 98.4%  (91.5% to 100%) | 32 | 100%  (89.1 %to 100%) | 30 | 96.8%  (83.3% to 99.9%) |
| T2 | 57 | 90.5%  (80.4% to 96.4%) | 29 | 90.6%  (75.0% to 98.0%) | 28 | 90.3%  (74.3% to 98.0% |
| T3 | 59 | 93.7%  (84.5 % to 98.2%) | 30 | 93.8%  (79.2% to 99.3%) | 29 | 93.5%  (78.6% to 99.2%) |

Table S3. Summary of qualitative interviews about reasons for laughter of 19 patients who noticed the laughter in the laughter task.

| Control group | CBM-pa group |
| --- | --- |
| Talking about something funny | Colleagues joking |
| Jokes they were telling | Cleaner was having a laugh. Try to wind up people with mental illness |
| I think its part of the study | See how I react |
| People on phone laughing or something they’ve seen. | If I be paranoid or upset |
| To see my response | To see how I react |
| Somebody got upset | It was computer generated |
| Some forces (not human) were having a laugh | Online thing that was funny |
| Laughing at seeing something | The reasons were to provoke a response from me |
| Someone making a joke | Student was showing you something. He played it [on phone] |
|  | To see how I felt about you being the laugh |

Table S4. Variance estimates for clinical outcomes at baseline with 80% and 95% confidence intervals (C.I). Highlighted are the upper 80% confidence intervals which are recommend using as an estimate for variance in future sample size calculations Confidence intervals are non-parametric bootstrap intervals based on 1000 samples with replacement.

| Variable | n | S.D. | 80 % C.I. | | 95% C.I. | |
| --- | --- | --- | --- | --- | --- | --- |
|  |  |  | Lower | Upper | Lower | Upper |
| SRT Target Bias score | 63 | 0.51 | 0.45 | **0.56** | 0.42 | 0.59 |
| SRT Foil Bias score | 63 | 0.44 | 0.38 | **0.51** | 0.34 | 0.54 |
| SST Bias score: Paranoia | 63 | 25.69 | 23.08 | **28.30** | 21.70 | 29.68 |
| SST Bias score: Non-Paranoia | 63 | 25.75 | 22.97 | **28.52** | 21.50 | 29.99 |
| Cognitive flexibility scale | 62 | 7.68 | 6.50 | **8.86** | 5.88 | 9.48 |
| PANSS (Positive) | 60 | 6.54 | 6.01 | **7.08** | 5.72 | 7.36 |
| PANSS (Negative) | 60 | 7.02 | 6.31 | **7.73** | 5.94 | 8.10 |
| PANSS (General) | 63 | 10.38 | 9.09 | **11.66** | 8.42 | 12.34 |
| PANSS (Total) | 57 | 19.03 | 16.73 | **21.32** | 15.51 | 22.54 |
| PANSS Item 6 | 63 | 0.98 | 0.89 | **1.06** | 0.84 | 1.11 |
| Paranoia Scale Total | 59 | 17.46 | 15.66 | **19.27** | 14.71 | 20.22 |
| Paranoid Thoughts Scale: Self reference | 59 | 16.52 | 14.74 | **18.30** | 13.80 | 19.24 |
| Paranoid Thoughts Scale: Persecution | 62 | 19.68 | 18.11 | **21.25** | 17.28 | 22.09 |
| Paranoid Thoughts Scale: Total | 58 | 35.90 | 32.19 | **39.60** | 30.23 | 41.56 |
| Peters Delusions Inventory | 61 | 5.45 | 4.93 | **5.98** | 4.65 | 6.26 |
| HADS: Anxiety | 61 | 4.37 | 3.90 | **4.84** | 3.65 | 5.08 |
| HADS: Depression | 62 | 3.72 | 3.28 | **4.15** | 3.06 | 4.38 |
| HADS: Total | 61 | 7.03 | 6.31 | **7.76** | 5.92 | 8.15 |

*Note*. SRT: Similarity Rating Task; SST: Scramble Sentences Task; HADS: Hospital Anxiety and Depression Scale; PANSS: Positive and Negative Symptom Scale

Table S5. Gender differences: Means, standard deviations and sample sizes for clinical outcomes at baseline (Time 0), post-treatment (Time 1), 1 month (Time 2) and 3 months (Time 3) within each treatment arm separately for males and females.

|  | | | **Males** | | **Females** | |
| --- | --- | --- | --- | --- | --- | --- |
| **Measure** | **Arm** | **Time** | **n** | **Mean (SD)** | **n** | **Mean (SD)** |
| Similarity Rating Task Target: Bias | CBM-pa | 0 | 22 | -0.28 (0.48) | 10 | -0.03 (0.7) |
|  | CBM-pa | 1 | 22 | -0.33 (0.5) | 8 | -0.08 (0.44) |
|  | CBM-pa | 2 | 21 | -0.51 (0.46) | 6 | -0.51 (0.76) |
|  | CBM-pa | 3 | 22 | -0.34 (0.47) | 7 | -0.32 (0.72) |
|  | Control | 0 | 20 | -0.2 (0.46) | 11 | -0.1 (0.46) |
|  | Control | 1 | 19 | -0.33 (0.71) | 10 | -0.84 (0.75) |
|  | Control | 2 | 18 | -0.74 (0.79) | 10 | -0.9 (0.79) |
|  | Control | 3 | 19 | -0.62 (0.92) | 10 | -0.89 (0.72) |
| Similarity Rating Task Foil: Bias | CBM-pa | 0 | 22 | -0.21 (0.39) | 10 | 0.17 (0.51) |
|  | CBM-pa | 1 | 22 | -0.15 (0.55) | 8 | -0.02 (0.4) |
|  | CBM-pa | 2 | 21 | -0.14 (0.36) | 6 | 0.03 (0.43) |
|  | CBM-pa | 3 | 22 | -0.13 (0.44) | 7 | -0.01 (0.37) |
|  | Control | 0 | 20 | -0.07 (0.44) | 11 | -0.05 (0.43) |
|  | Control | 1 | 19 | -0.18 (0.52) | 10 | -0.27 (0.27) |
|  | Control | 2 | 18 | -0.14 (0.54) | 10 | -0.29 (0.32) |
|  | Control | 3 | 19 | -0.19 (0.64) | 10 | -0.16 (0.17) |
| Scrambled Sentences Task: Paranoid bias | CBM-pa | 0 | 22 | 38.37 (29.05) | 10 | 49.6 (28.56) |
|  | CBM-pa | 1 | 21 | 48.16 (22.96) | 8 | 48.12 (22.49) |
|  | Control | 0 | 20 | 31.63 (23.54) | 11 | 42.25 (17.34) |
|  | Control | 1 | 19 | 39.74 (41.02) | 10 | 23.25 (17.56) |
| Scrambled Sentences Task: Non-paranoid bias | CBM-pa | 0 | 22 | 34.93 (31.27) | 10 | 30.4 (21.05) |
|  | CBM-pa | 1 | 21 | 31.58 (22.32) | 8 | 30.92 (24.01) |
|  | Control | 0 | 20 | 38.69 (25.18) | 11 | 45.68 (17.65) |
|  | Control | 1 | 19 | 51.61 (27.11) | 10 | 62.97 (29.69) |
| Cognitive flexibility | CBM-pa | 0 | 22 | 46.91 (5.34) | 10 | 45.3 (12) |
|  | CBM-pa | 1 | 19 | 47.26 (7.1) | 8 | 48.63 (9.35) |
|  | Control | 0 | 20 | 45.85 (6.83) | 10 | 50.1 (8.61) |
|  | Control | 1 | 19 | 45.16 (6.22) | 9 | 54.78 (29.91) |
| PANSS: Item 6 | CBM-pa | 0 | 22 | 5.05 (1.05) | 10 | 5.5 (0.71) |
|  | CBM-pa | 1 | 21 | 4.19 (1.4) | 8 | 4.5 (1.31) |
|  | CBM-pa | 2 | 21 | 4.48 (1.4) | 7 | 4.71 (1.38) |
|  | CBM-pa | 3 | 21 | 4.29 (1.59) | 8 | 5.38 (1.77) |
|  | Control | 0 | 20 | 5 (0.97) | 11 | 5.18 (1.08) |
|  | Control | 1 | 19 | 4.16 (1.38) | 10 | 3.9 (1.73) |
|  | Control | 2 | 18 | 4.39 (1.24) | 10 | 4.1 (1.6) |
|  | Control | 3 | 19 | 3.95 (1.75) | 10 | 4.3 (1.95) |
| Paranoia scale | CBM-pa | 0 | 20 | 54.05 (15.14) | 10 | 77.7 (11.81) |
|  | CBM-pa | 1 | 19 | 56.11 (19.55) | 8 | 69.75 (17.76) |
|  | CBM-pa | 2 | 20 | 50.75 (17.16) | 5 | 70.8 (14.75) |
|  | CBM-pa | 3 | 21 | 52.14 (14.98) | 8 | 65 (17) |
|  | Control | 0 | 18 | 60.27 (18.58) | 11 | 67.91 (14.36) |
|  | Control | 1 | 18 | 51.56 (17.3) | 9 | 61 (16.89) |
|  | Control | 2 | 18 | 49.11 (14.45) | 7 | 42.86 (13.59) |
|  | Control | 3 | 18 | 51.78 (15.71) | 9 | 53.11 (15.31) |
| Paranoid Thoughts Scale: Total | CBM-pa | 0 | 19 | 84.47 (30.53) | 10 | 114 (30.91) |
|  | CBM-pa | 1 | 18 | 78.72 (38.47) | 7 | 98 (42.64) |
|  | CBM-pa | 2 | 21 | 79.62 (39.24) | 5 | 94 (45.03) |
|  | CBM-pa | 3 | 21 | 77.19 (39) | 8 | 97.38 (37.73) |
|  | Control | 0 | 19 | 79.32 (32.98) | 10 | 93.9 (46.97) |
|  | Control | 1 | 19 | 65.26 (35.54) | 9 | 87.78 (47.89) |
|  | Control | 2 | 18 | 66.5 (29.44) | 10 | 72.3 (40.29) |
|  | Control | 3 | 19 | 66.42 (34.14) | 9 | 79.67 (45.05) |
| Peters Delusions Inventory | CBM-pa | 0 | 22 | 8.68 (5.33) | 10 | 11.9 (4.09) |
|  | CBM-pa | 1 | 20 | 7.8 (4.42) | 7 | 9.57 (4.12) |
|  | CBM-pa | 2 | 0 | . (.) | 0 | . (.) |
|  | CBM-pa | 3 | 14 | 7.29 (4.61) | 6 | 8.33 (6.19) |
|  | Control | 0 | 20 | 9.6 (6.71) | 9 | 9 (3.67) |
|  | Control | 1 | 17 | 8.12 (5.45) | 9 | 10.67 (8.32) |
|  | Control | 2 | 0 | . (.) | 0 | . (.) |
|  | Control | 3 | 13 | 7.54 (5.91) | 9 | 7.56 (6.39) |
| HADS: Total score | CBM-pa | 0 | 21 | 16.38 (6.21) | 10 | 21.1 (7.87) |
|  | CBM-pa | 1 | 21 | 16.67 (7.19) | 8 | 21 (9.12) |
|  | CBM-pa | 2 | 21 | 17.05 (8.21) | 7 | 17.57 (8.6) |
|  | CBM-pa | 3 | 20 | 15.95 (6.97) | 8 | 21.13 (10.87) |
|  | Control | 0 | 20 | 19.65 (7.47) | 10 | 21.1 (6.14) |
|  | Control | 1 | 19 | 17.58 (6.83) | 10 | 17.7 (7.18) |
|  | Control | 2 | 18 | 17.83 (6.64) | 10 | 17.5 (8.76) |
|  | Control | 3 | 18 | 17.89 (8.22) | 10 | 19.1 (10.71) |
| VRE pre –post change: Anxiety | CBM-pa | 0 | 21 | 39.29 (28.65) | 8 | 49.13 (30.9) |
|  | CBM-pa | 1 | 21 | 28.81 (23.29) | 8 | 48.75 (27.48) |
|  | Control | 0 | 18 | 40 (25.67) | 10 | 44.5 (30.95) |
|  | Control | 1 | 19 | 31.58 (18.86) | 10 | 36.5 (33.83) |
| VRE pre –post change: Sadness | CBM-pa | 0 | 21 | 28.33 (28.17) | 8 | 32.25 (26.44) |
|  | CBM-pa | 1 | 21 | 31.67 (28.87) | 8 | 28.75 (24.75) |
|  | Control | 0 | 18 | 22.5 (17.59) | 10 | 30 (25.39) |
|  | Control | 1 | 19 | 23.42 (17.88) | 10 | 19.5 (24.32) |
| VRE pre –post change: Paranoia | CBM-pa | 0 | 21 | 22.29 (22.4) | 8 | 33.13 (28.65) |
|  | CBM-pa | 1 | 21 | 24.05 (28.62) | 8 | 36.88 (31.95) |
|  | Control | 0 | 18 | 30.83 (24.03) | 10 | 42 (29.08) |
|  | Control | 1 | 19 | 36.58 (27.49) | 10 | 31.5 (33.67) |
| VRE pre –post change: Friendly | CBM-pa | 0 | 21 | 73.1 (19.97) | 8 | 50 (28.78) |
|  | CBM-pa | 1 | 21 | 69.67 (27.94) | 8 | 56.25 (31.59) |
|  | Control | 0 | 18 | 57.78 (21.78) | 10 | 70.5 (26.71) |
|  | Control | 1 | 19 | 58.16 (25.94) | 10 | 58 (30.75) |
