# Supplementary material for: Cognitive bias modification for paranoia (CBM-pa): a randomised controlled feasibility study in patients with distressing paranoid beliefs
Source: Psychol Med. 2022 Jun 14;53(10):4614–26. doi: 10.1017/S0033291722001520 (PMC10388312; doi:10.1017/S0033291722001520)
Supplement: Supplementary file 1 [file S0033291722001520sup.zip › S0033291722001520sup002.docx]

**Cognitive bias modification for paranoia (CBM-pa): A randomised controlled feasibility study in patients with distressing paranoid beliefs**

**Supplementary materials**

**Measures of clinical symptoms**

*Paranoia Scale* (PS; Fenigstein & Vanable, 1992) was a 20-item self-report scale measuring different aspects of paranoid thoughts in the present study. Participants rated their responses on a 5-point Likert scale ranging from 1*, not at all applicable to me*, to 5, *extremely applicable to me*. The higher the scores, the greater the paranoid ideation (ranging from 20 to 100). In the current study, it had a high degree of internal consistency (Cronbach’s α = 0.92).

*The Green et al Paranoid Thoughts Scale* (GPTS; Green et al., 2008)*,* comprising two 16-item scales, was used to measure ideas of social reference and ideas of persecution. Each item was rated on a five-point Likert scale. Higher scores reflect greater levels of paranoid thinking (range is from 16-80). The GPTS had a high internal consistency in the current study (Cronbach’s α = 0.97).

*Positive and Negative Symptom Scale* (PANSS; Kay et al., 1987) was administered by a trained clinical psychologist at baseline to provide diagnostic information to characterise the sample. The PANSS item 6 (P6 Suspiciousness/Persecution) was used alone at post-treatment (T1), interim sessions (I1, I2, I3, I4) and follow-ups (T2, T3) as a clinical measure of paranoia. This item measures unrealistic or exaggerated ideas of persecution using a 7-point Likert scale from *1* = *absent* to 7 = *extreme persecutory delusions*.

*The Peters et al Delusion Inventory* (PDI-21, Peters et al., 2004) was administered to assess delusional symptoms. Participants rated their responses in a yes/no format. Participants answering “yes” to any questions then rated the degree of conviction, preoccupation and distress using a 5-point Likert scale. The higher the scores, the greater degree of delusional ideation. The PDI-21 yielded a Cronbach’s α of 0.88 for the total score.

*Hospital Anxiety and Depression Scale* (HADS; Zigmond & Snaith, 1983). The HADS, consisting of 14 items, was used to measure symptoms of clinical anxiety and depression. Each item was scored from 0 to 3. The higher the scores, the greater the mood disturbance. The HADS was shown to possess good internal reliability in this sample (Cronbach’s α = 0.72 and 0.83 for depression and anxiety subscales respectively).

*Cognitive Flexibility Scale* (CFS; Martin & Rubin, 1995) was used to measure cognitive flexibility. Paranoid traits in the general population are associated with reduced cognitive flexibility (Freeman et al., 2008) and reduced cognitive flexibility has been found to predict paranoid interpretation bias in a healthy sample (Savulich et al., 2015). Since cognitive flexibility reflects the ability to consider different alternatives it could be an important mediator of responsiveness to the CBM-pa intervention and/or could itself be altered by an intervention such as CBM-pa which provides repeated practice in manipulating cognitive processing. Savulich et al., (2015) suggest it should be routinely measured when cognitive biases are either assessed or manipulated. CFS consisted of 12 items. Participants rated on a 6-point scale ranging from 1 (*strongly disagree*) to 6 (*strongly agree*). The higher the scores, the greater the cognitive flexibility. It had a good internal consistency (Cronbach’s α = 0.69).

*Measure of Stress/ Distress*

*Laughter task* (Green et al., 2011). Two experimental events were presented during the testing session to simulate two ‘real-life’ events that were known to elicit paranoid interpretations in the general population: (a) an interruption by a stooge calling the experimenter out of the room and (b) following the exit of the stooge and the experimenter, a 35s audio recording of laughter played outside the room. Participants were asked to assess their interpretations and emotional responses to these two events. Verbatim explanations given by the participants were rated for the presence of a paranoid attribution.

*Virtual Reality Environment* (VRE). Participants wore a headset through which they viewed a three- dimensional social scene populated by neutral characters (a café) for approximately four minutes. A series of VAS ratings asking how anxious, sad, paranoid and friendly participants felt before and after experiencing the virtual environment was obtained. Participants also completed the *State Social Paranoia Scale* (SSPS; Freeman et al., 2007), a measure of recent paranoid thinking. Each item was scored on a 5-point Likert scale (*Do not agree* – *Totally agree*). Higher scores indicate greater levels of persecutory thinking.
